# QUARTERplus: Accurate disorder predictions integrated with interpretable residue-level quality assessment scores

**DOI:** 10.1016/j.csbj.2021.04.066

**Published:** 2021-04-27

**Authors:** Akila Katuwawala, Sina Ghadermarzi, Gang Hu, Zhonghua Wu, Lukasz Kurgan

**Affiliations:** a Department of Computer Science, Virginia Commonwealth University, Richmond, VA 23284, USA; b School of Statistics and Data Science, LPMC and KLMDASR, Nankai University, Tianjin 300071, China; c School of Mathematical Sciences and LPMC, Nankai University, Tianjin 300071, China

**Keywords:** Intrinsic disorder, Disorder prediction, Quality assessment, Deep learning, Meta-prediction, Webserver

## Abstract

A recent advance in the disorder prediction field is the development of the quality assessment (QA) scores. QA scores complement the propensities produced by the disorder predictors by identifying regions where these predictions are more likely to be correct. We develop, empirically test and release a new QA tool, QUARTERplus, that addresses several key drawbacks of the current QA method, QUARTER. QUARTERplus is the first solution that utilizes QA scores and the associated input disorder predictions to produce very accurate disorder predictions with the help of a modern deep learning meta-model. The deep neural network utilizes the QA scores to identify and fix the regions where the original/input disorder predictions are poor. More importantly, the accurate QUATERplus’s predictions are accompanied by easy to interpret residue-level QA scores that reliably quantify their residue-level predictive quality. We provide these interpretable QA scores for QUARTERplus and 10 other popular disorder predictors. Empirical tests on a large and independent (low similarity) test dataset show that QUARTERplus predictions secure AUC = 0.93 and are statistically more accurate than the results of twelve state-of-the-art disorder predictors. We also demonstrate that the new QA scores produced by QUARTERplus are highly correlated with the actual predictive quality and that they can be effectively used to identify regions of correct disorder predictions. This feature empowers the users to easily identify which parts of the predictions generated by the modern disorder predictors are more trustworthy. QUARTERplus is available as a convenient webserver at http://biomine.cs.vcu.edu/servers/QUARTERplus/.

## 1 Introduction

Proteins and protein sequence regions that lack a stable three-dimensional structure under physiological conditions are referred to as intrinsically disordered proteins (IDPs) and intrinsically disordered regions (IDRs), respectively [1], [2], [3]. They play key roles for a wide spectrum of cellular functions that include signaling, cell cycle regulation, transcription, translation, splicing, and post transitional modifications [4], [5], [6], [7]. Computational studies suggest that IDPs and proteins with IDRs are highly abundant across all domains of life, with as many as 30% of eukaryotic proteins that are projected to have long IDRs (30 or more consecutive amino acids) [8], [9]. Motivated by the fact that IDRs possess unique sequence signatures, dozens of computational tools that predict intrinsic disorder from protein sequences were developed during the last two decades [10], [11], [12], [13], [14], [15], [16], [17]. These methods find a variety of applications that include investigations that focus on structural and functional characterization of individual proteins and protein families [18], [19], [20] and broad-scale studies that analyze prevalence and functional roles of disorder over and across genomes [9], [21], [22], [23], [24], [25], [26], [27]. The above studies rely on the availability of accurate disorder predictors. Past comparative assessments demonstrate that some disorder predictors provide accurate results [11], [13], [28], [29], [30], [31], [32], [10], [33]. While these assessments use datasets composed of dozens or hundreds of proteins to provide insights into an overall predictive performance of these tools, they do not offer guidance when dealing with predictions for specific proteins. A recent study reveals that performance of even the best predictors varies widely between proteins, where some proteins are predicted exceptionally well while other predictions are barely better than random [34]. Other studies find that the quality of the disorder predictions varies between amino acids in the same protein and that the outputs produced by the predictors do not allow to accurately identify these differences [35], [36].

We devised first-of-its-kind solution to these problems in 2019, with the release of the QUARTER tool that generates quality assessment (QA) scores for the disorder predictions [36], [37]. This work was originally inspired by the intensely researched QA for the putative tertiary protein structures [38], [39], [40], [41], [42], [43], but here we quantify the quality of the disorder predictions at the residue level. The QA scores quantify correctness (confidence) of the disorder predictions at a residue level to reveal which predictions produced by a given tool are more likely to be correct [36]. In other words, users can utilize the QA scores to accurately identify residues in a given protein chain for which the disorder predictions are more likely to be accurate. We developed the QUARTER’s QA scores for ten popular disorder predictors [36], [37]. The research behind QUARTER demonstrates that the QA scores produced directly from the propensities of intrinsic disorder generated by these ten methods have poor quality, motivating the need for new tools that produce high-quality QA scores [35]. However, QUARTER, which is the only tool capable of producing accurate QA scores, suffers three substantial drawbacks. **First**, the QA scores produced by QUARTER are difficult to interpret as they do not represent a specific metric. This means that while higher QA values identify relatively better predictions, it is difficult to judge whether these high scores in fact correspond to high-quality results. For instance, high scores could correspond to exceptionally accurate predictions compared to lower scores that denote (relatively worse) accurate predictions, in which case the lower scores may give a false impression that the corresponding residues are predicted poorly. **Second**, the QUARTER-produced QA scores were so far used only to identify quality of the disorder predictions while they could be used to correct and consequently improve these predictions. **Third**, the original implementation makes it difficult to apply QUARTER since it requires the disorder predictions generated with a third-party software/server as inputs. This way the users have to go through two separate steps (predict disorder and next predict QA scores) to secure the complete results.

We address these three major issues with the new QUARTERplus platform that provides substantially improved disorder predictions together with interpretable residue-level QA scores. More specifically, QUARTERplus offers:

• High-quality disorder predictions based on a novel deep learning meta-model that combines QA scores and the associated predictions from three popular disorder predictors. This is the first time when a meta-model uses QA scores to outperform a representative collection of modern disorder predictors.
• Easy to interpret residue-level QA scores that quantify quality of the predictions for ten popular disorder predictors, including the novel meta-model.
• Webserver implementation that conveniently automates the prediction of disorder and generation of the interpretable QA scores directly from an input amino acid sequence.

## 2 Materials and methods

### 2.1 Datasets

We obtain the source data to derive training and test datasets from a recent large-scale assessment study [31]. The source dataset includes 25,717 proteins with the native intrinsic disorder annotations that were collected from the MobiDB database [44]. Following the QUARTER article that relies on the same source data [36], we refine these data to accommodate for the needs of the subsequently used tools and the test standards in this area. In particular, we ensure a proper level of separation (i.e., low levels of the sequence similarity) between training and test datasets [13]. First, we remove sequences with non-standard amino acids. Next, we reduce the pairwise sequence similarity among the remaining 12,129 sequences to below 25% using BLASTCLUST [45]. The resulting dataset consists of 6272 proteins with 105,709 disordered and 1,672,907 ordered residues. We divide this protein set at random into the test set with 999 proteins and training set with 5271 proteins. The two datasets are available at http://biomine.cs.vcu.edu/servers/QUARTERplus/. We use the training dataset to design and optimize QUARTERplus, i.e., to empirically map the raw QUARTER’s QA scores into novel interpretable QA scores and to train and parametrize the novel meta-predictor of disorder. We utilize the independent test dataset to comparatively assess QUARTERplus. The independence of this test set stems from its low, <25% similarity to the training dataset and the fact that it was excluded from the training process.

### 2.2 Selection of disorder predictors

We rationally select a representative set of disorder predictors that we outfit with the QUARTERplus-derived QA scores and compare with the new meta disorder predictor. We start with the list of methods that were included in a recent large-scale (i.e., using large dataset) comparative study [31]. We exclude three tools from that study, namely SEG [46], Pfilt [47] and FoldIndex [48], given their relatively poor predictive quality reported there [31]. We consider the remaining ten disorder predictors including DisEMBL‐465 (trained using X‐ray structures) and DisEMBL‐HL (trained to predict disorder-like loop conformations), which are two versions of the DisEMBL predictor [49]; three versions of ESpritz [50]: ESpritz‐Xray (trained on X‐ray structures), ESpritz‐NMR (trained on NMR structures) and ESpritz‐DisProt (trained on data from the DisProt database [51]); two flavors of IUPred [52], [53]: IUPred‐short (trained to predict short IDRs) and IUPred‐long (trained to predict long IDRs); GlobPlot [54]; RONN[55] and VSL2B [56]. Moreover, we extend this set of ten predictors guided by the results from recent and smaller scale (i.e., using smaller datasets) assessments. Consequently, we add the popular DISOPRED3 [57] that was among the top ranked methods in the CASP10 assessment [29], the last CASP (Critical Assessment of protein Structure Prediction) that included the disorder prediction. We also include SPOT-Disorder [58], a deep neural network that was ranked among the best published predictors in the most recent CAID (Critical Assessment of protein Intrinsic Disorder prediction) [33]. The resulting set of twelve tools includes VSL2B, SPOT-Disorder and ESpritz-Disprot that secured the top-three results in another recent assessment [13]. Finally, in keeping with the CASP tradition we exclude the methods that were developed by the authors of this article.

We collect disorder predictions for the ten methods that were covered in [31] directly from MobiDB [44]. We obtain predictions for the remaining two predictors (DISOPRED3 and SPOT-Disorder) that are not included in that database using their standalone software provided with the publications. We generate the QUARTER’s QA scores using its webserver [36].

### 2.3 Assessment of predictive performance

QUARTERplus produces two outputs: the QA scores and the disorder predictions. The disorder predictions consist of a real-valued propensity that quantifies likelihood that a given amino acid is disordered and a corresponding binary classification (disordered vs. structured). Typically, the binary predictions are generated from the propensities, such that residues with propensities greater than a given threshold are predicted as disordered, and otherwise they are predicted as structured. We use the area under the receiver-operating characteristic curve (ROC-AUC) as the primary metric to evaluate predictive quality of the propensities. ROC-AUC is arguably the most commonly used measure for the assessment of the disorder predictors [11], [12], [29], [31], [34], [36]. ROC curve is a relation between true-positive rates (TPRs) and false-positive rates (FPRs) that is computed by thresholding the propensities where the thresholds are the set of all unique propensities produced by a given predictor. ROC-AUC ranges between 0.5 (equivalent to a random prediction) and 1 (perfect prediction). We also report values of the area under the precision-recall curve (PR-AUC) to provide a secondary evaluation of the predicted propensities. PR-AUC ranges between 0 (random predictor) and 1 (perfect predictor). We assess the binary predictions with the Matthews Correlation Coefficient (MCC) that ranges between −1 and 1, where −1 denotes an inverted prediction (all predictions are flipped compared to the experimental values), 0 denotes a random result and 1 denotes a perfect prediction.

We also measure the quality of prediction of the QA scores produced by QUARTERplus for a given disorder predictor. We compare these residue-level scores against the true predictive quality secured by this predictor using the Pearson’s correlation coefficient (PCC), mean squared error (MSE) and Mean absolute error (MAE).

### 2.4 Quarterplus architecture

QUARTERplus applies an innovative approach to produce very accurate disorder predictions that are accompanied by new interpretable QA scores. We integrate the QUARTER-produced QA scores into a proven meta-predictor approach that was demonstrated to produce highly accurate disorder predictions [59], [60], [61], [62], [63], [64], [65]. The past meta-predictors rely on shallow predictive models including neural networks [59], [65], regression [61], support vector machine [62], boosted decision trees [63], and simple scoring functions [60], [64]. Here, we use a more modern deep network to combine multiple disorder predictions that are accompanied, for the first time, by the QA scores. The underlying premise is that the deep network utilizes the QA scores to identify residues that are poorly predicted in a specific input disorder prediction, which then can be “repaired” with the help of the other disorder predictions.

**Fig. 1:**
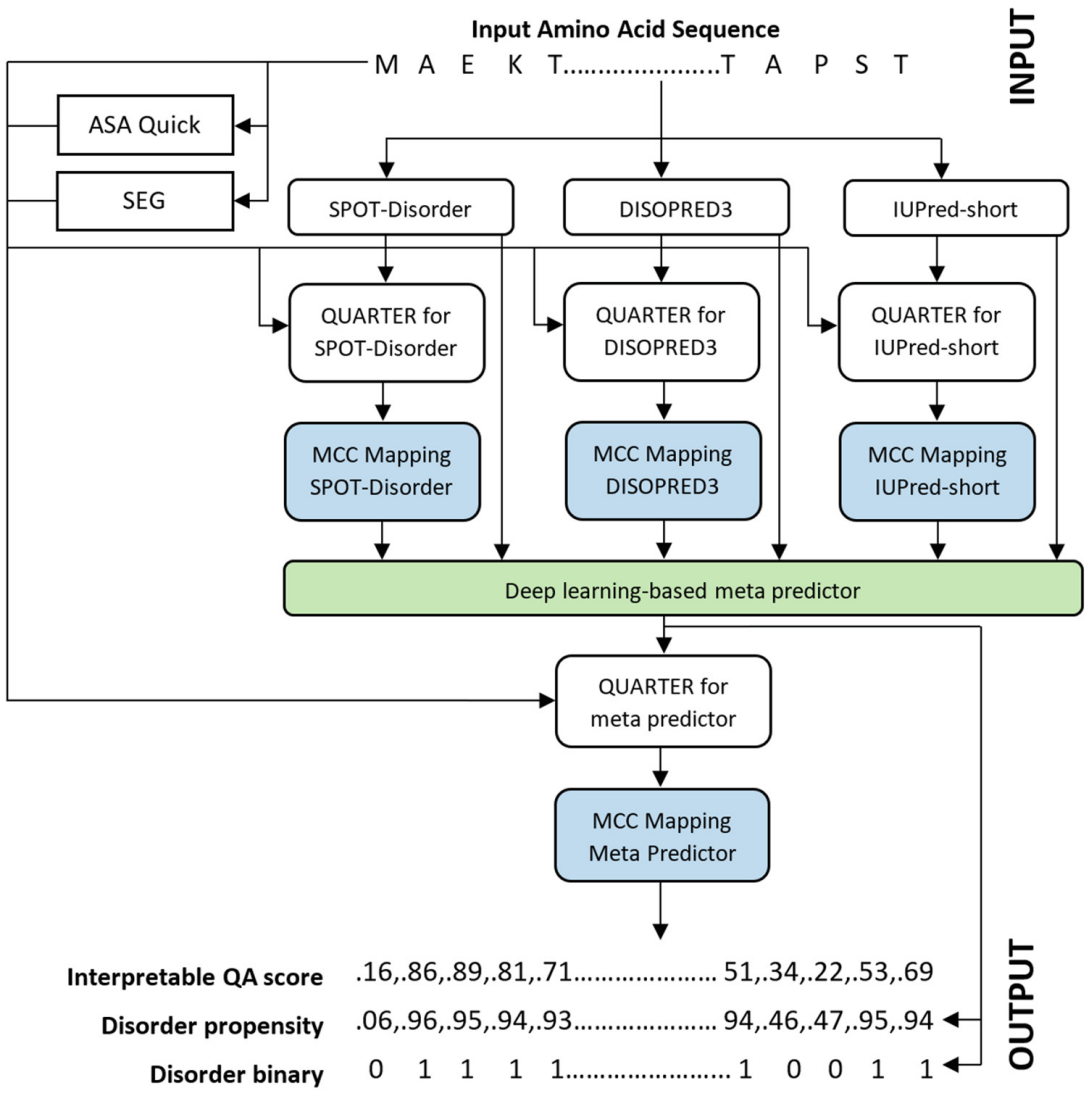
Architecture of the QUARTERplus method. The input amino acid sequence is used to predict QA scores that reflect MCC values (shown in blue). QUARTERplus outputs the disorder predictions (propensities and binary) produced by the meta-predictor implemented with deep neural network (shown in green) which are accompanied with the QA scores. (For interpretation of the references to color in this figure legend, the reader is referred to the web version of this article.)

We summarize the QUARTERplus architecture in Fig. 1. We use the input amino acid sequence to make disorder predictions with SPOT‐Disorder [58], DISOPRED3 [57] and IUPred‐short [52]. Next, we process their outputs with QUARTER to produce raw QA scores. We map the raw scores into the new interpretable QA scores and feed them together with the corresponding disorder predictions into the meta-predictor implemented with a deep neural network. We utilize this meta model to produce improved disorder predictions that we further process with the QUARTER and the mapping function to produce the interpretable QA scores. The final outputs include the disorder predictions (propensities and binary values) and the accompanying residue-level QA scores. We discuss details of the mapping and the meta architecture in the subsequent sections.

### 2.5 Interpretable QA scores

The original/raw QA scores produced by QUARTER are difficult to interpret. We overcome this issue by mapping the raw QA scores into intuitive scores that are normalized to correspond to MCC values. We select MCC since it is one of the most popular metrics for the disorder predictions [11], [12], [29], [31], [34], [36], is easy to interpret (i.e., the values can be intuitively understood) and because its fixed range of values is more expressive than other commonly used metrics. More specifically, the MCCs values expresses multiple scenarios where the prediction is expected to be correct (high positive MCC), to have poor quality (near zero MCC) and to be flipped (high negative MCC).

**Fig. 2:**
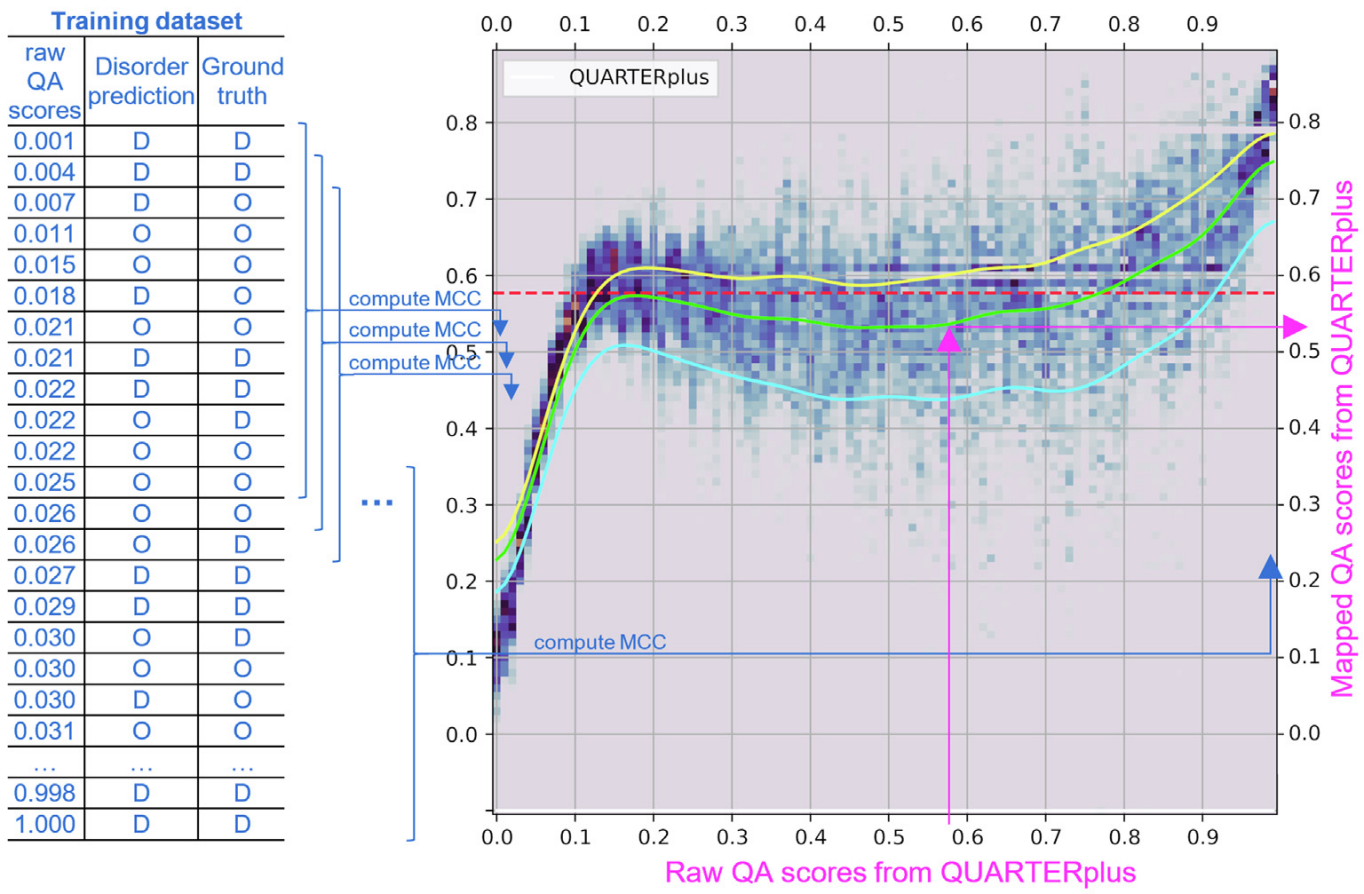
MCC mapping functions that transform the raw QUARTER-produced QA scores into the interpretable QA scores. The blue elements on the left explain calculation of the MCC distributions that are used to derive the mapping. We sort the raw residue-level QA scores generated by QUARTER for a given disorder predictor in the training dataset to calculate MCC for a collection of residues covered by a sliding window. The MCC is computed by comparing the predicted and ground truth disorder in that window. We use these MCC scores to derive the mapping function shown in the plot. While this plot is specific to the QUARTERplus meta predictor, we provide the complete set of 13 mappings for all considered disorder predictors is in Supplementary Fig. S1. The yellow, green and blue lines correspond to 75th, 50th (median) and 25th percentile of the MCC scores computed using the sliding window. The red horizontal dashed line is the MCC obtained on the training dataset. The pink elements show how the raw QA scores and converted to the mapped QA scored with the help of the green median line. (For interpretation of the references to color in this figure legend, the reader is referred to the web version of this article.)

We derive the MCC mapping function exclusively using the training dataset (Fig. 2). First, we sort the raw residue-level QA scores generated by QUARTER for a given disorder predictor. Next, we calculate MCC for a collection of residues covered by a sliding window over the sorted list of residues to represent the mapping for the residues in the middle of a given window (blue elements in Fig. 2). The plot in Fig. 2 illustrates the resulting relation between the MCC scores and the corresponding raw QA values for the QUARTERplus disorder predictor. The corresponding plots for the complete set of the considered 12 disorder predictors are shown in Supplementary Fig. S1. The green lines approximate the MCC mapping functions that we use to map the raw QA scores into the interpretable (MCC-like) QA scores (pink elements in Fig. 2).

Fig. 2 reveals that the mapped/interpretable QA scores (quantified with the MCC values) are correlated with the raw QA scores. This can be observed using the green lines that represent the mapped QA scores which increase as the values of the raw QA scores, given in the *x*-axis, increase (Supplementary Fig. S1). This is expected since both the raw and the mapped QA scores quantify the underlying quality of the disorder predictions. Importantly, while the mapped QA scores can be directly understood as they quantify correlation coefficients (i.e., MCC), the fact that the green line is non-linear and non-monotonic suggests that the raw QA scores are not interpretable. For example, the middle part of the relation for QUARTERplus is flat (see the green plot in Fig. 2). This means that the raw QA scores between 0.2 and 0.7 in fact correspond to virtually identical predictive performance while in practice they would be incorrectly interpreted as suggesting a substantial difference in quality. The mapped QA scores fix that issue since they directly correspond to the measured performance. We observe that the MCC mappings differ substantially across the disorder predictors (Supplementary Fig. S1). This means that their raw QA scores would have to be understood in very different ways, while the new mapped QA scores standardize the interpretation across this diverse set of predictors.

### 2.6 Deep learning-based meta predictor

Fig. 1 outlines the QUATERplus’s meta-design. First, we collect putative solvent accessibility predicted from the input sequence with ASAquick [66] and sequence complexity annotation produced from the sequence with the SEG algorithm [46]. Concurrently, we generate disorder predictions using SPOT‐Disorder [58], DISOPRED3 [57] and IUPred‐short [52]. This selection is motivated by the fact that these three methods secure the highest predictive performance on the training dataset and the desire to minimize the computational costs, which would increase if we would include additional methods. We need a minimum of three predictors since the odd number allows to break ties between conflicting predictions. Second, we input the disorder predictions together with the sequence complexity and putative solvent accessibility into QUARTER that generates the corresponding raw QA scores. Third, we map the raw QA scores into the novel interpretable QA scores. Next, we input the three disorder predictions and the corresponding three mapped QA scores into a deep neural network (green box in Fig. 1) that produces the disorder predictions. Finally, we process these predictions by QUARTER and map its outputs to collect the final interpretable QA scores that accompany the QUARTERplus-produced disorder predictions.

**Fig. 3:**
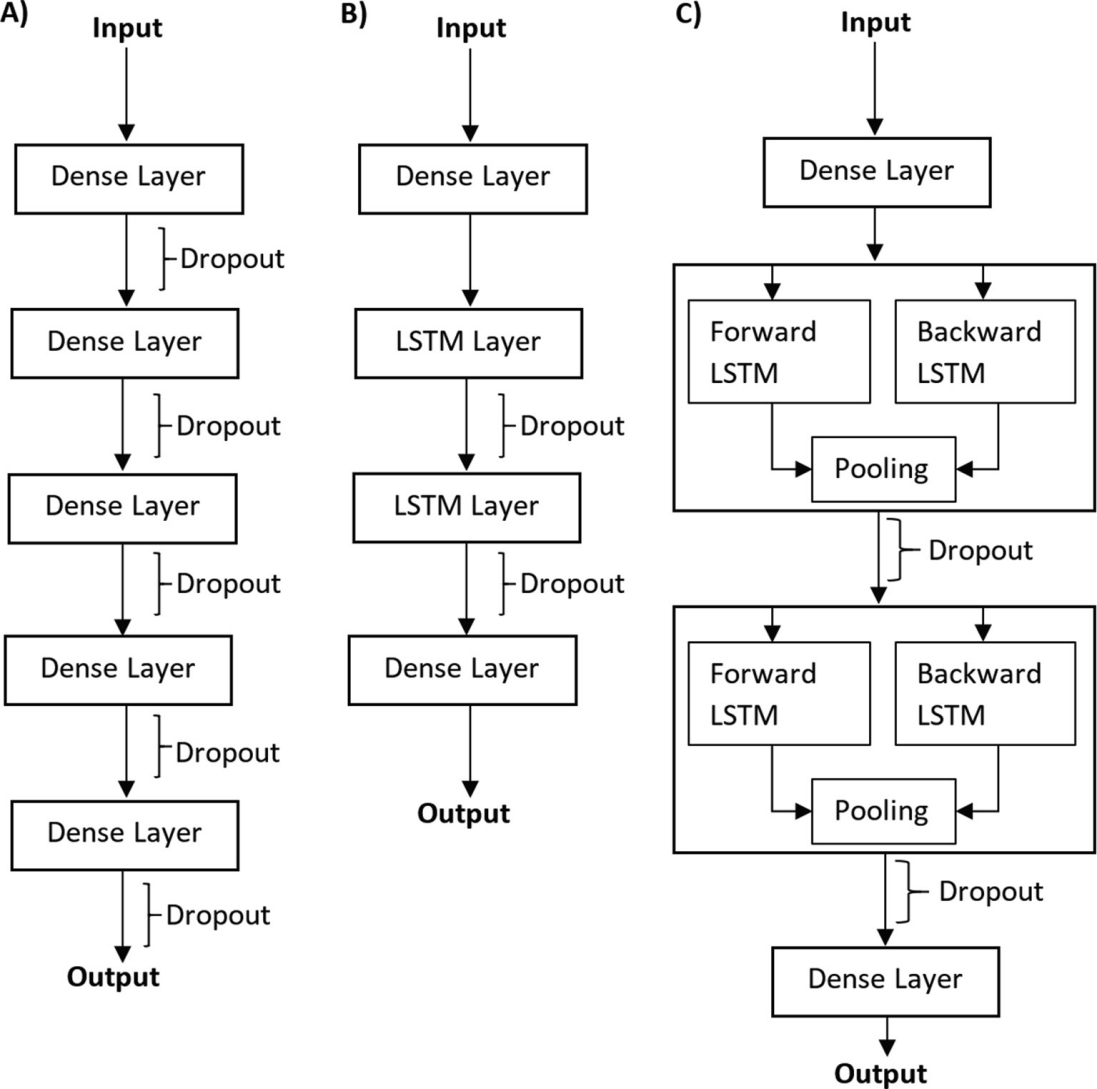
Deep neural network architectures that were considered to develop the QUARTERplus meta predictor of disorder. (A) Classical feed forward network with dropout in the intermediate layers. (B) Long-Short Term Memory (LSTM) network. (C) Modified LSTM network with bidirectional layers.

We empirically test and parametrize several types of modern deep networks (Fig. 3) to select the design that maximizes predictive performance on the training dataset. Our first choice was inspired by a state-of-the-art deep network disorder predictor SPOT-Disorder-Single [67] that was highly-ranked in CAID [33]. This architecture, shown in Fig. 3B, is a recurrent convolutional network that utilizes Long-Short Term Memory (LSTM) units. We further improved this design (Fig. 3C) by replacing the LSTM layers with arguably more advanced bidirectional LSTM layers. We also use a classical deep feedforward network (Fig. 3A) as a reference point that allows us to assess whether the more advanced recurrent convolutional designs provide the expected benefits. We parametrize each architecture by adjusting the size of neural layers, using the published 200 neurons per LSTM layers size as a starting point [67]. We gradually scale this large network down to 100, 50, 25, 12, 6 and 3 neurons per layer and use the dropout rate of 0.5 to minimize likelihood of overfitting into the training dataset [68]. Supplementary Table S1 details the considered 21 architectures (3 network types and 7 sizes). We subdivide the training dataset at random into the design and validation subsets. We train the networks on the design subset and assess the predictions of these trained models on the validation dataset to select the best alternative, i.e., the design with the highest ROC-AUC value. The training relies on 0.01 learning rate and decay rate of 1% over 50 epochs (defaults in the Keras library) and the loss function implemented with binary cross entropy [69]. Supplementary Fig. S2 compares results secured by the different deep networks on the validation set. As expected, we observe that the traditional feedforward networks are consistently outperformed by the convolutional topologies. We also find that the best results for the convolutional network are obtained with the mid-size networks. The best option is the modest size bi-directional LSTM design with 10 neurons in the dense layer and 25 neurons in the LSTM layers. This is the design that we use to implement QUARTERplus.

Finally, we also experiment with two popular shallow machine learning algorithms, logistic regression and k-nearest neighbor. The regression-based solution simulates a simple meta-predictor that relies on a weighted (by the coefficients on the regression function) average of the disorder predictions. After parametrization on the training dataset, these models obtain ROC-AUC = 0.907 (logistic regression) and 0.870 (nearest neighbor) on the test dataset, compared to 0.929 for the deep network. This further justifies the selection of the deep network model as the predictive engine.

## 3 Results

QUARTERplus generates disorder predictions that are accompanied by the interpretable QA scores. We empirically assess quality of both outputs using the independent test dataset which shares low (<25%) similarity with the training proteins.

### 3.1 Assessment of the interpretable QA scores

**Fig. 4:**
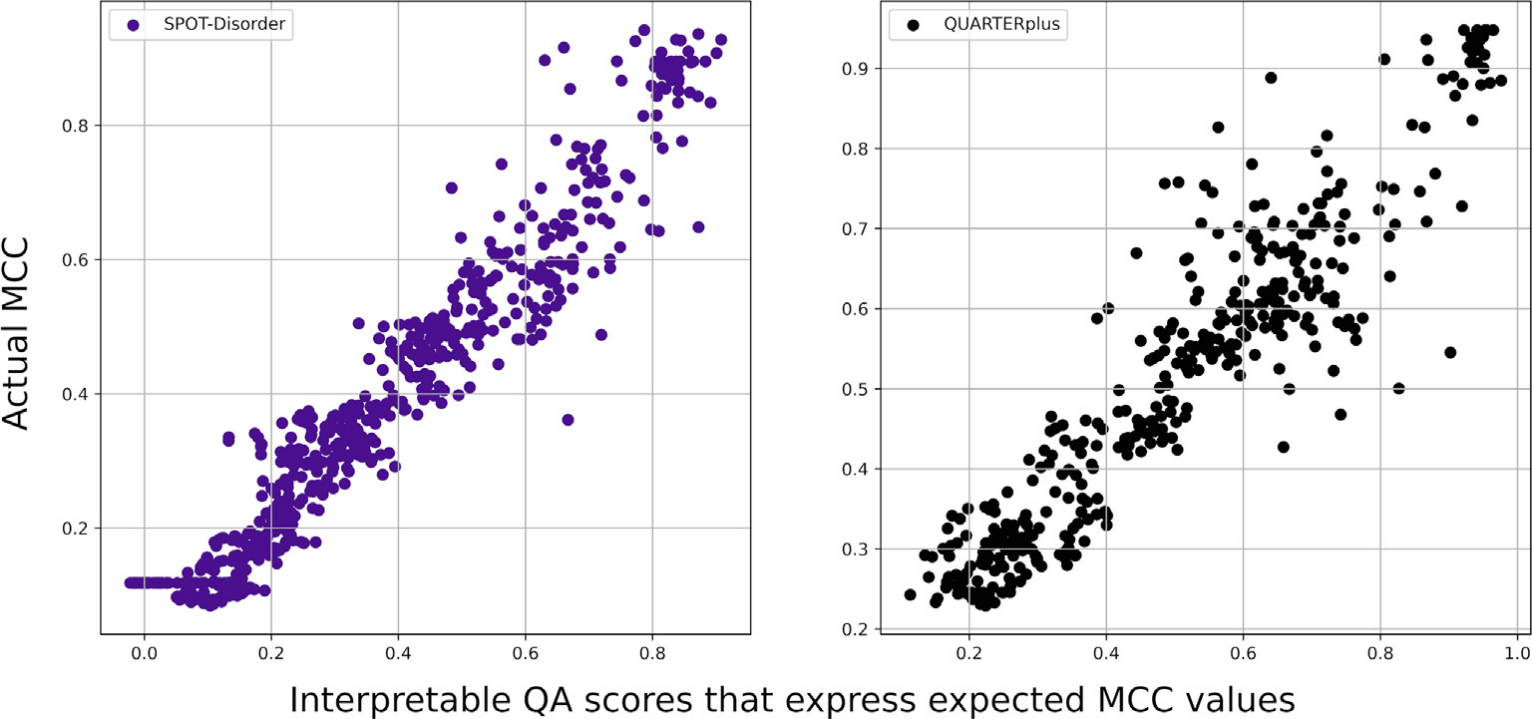
Scatter plots of the actual MCC values (*y*-axis) against the new QA scores that express expected MCC values (*x*-axis) computed on the independent test dataset for the SPOT‐Disorder and QUARTERplus disorder predictors. We use the sliding windows of the residues sorted by the QUARTER scores to produce the actual MCC scores (details in Section 2.5). The complete set of 13 distributions for all considered disorder predictors are in Supplementary Fig. S3.

We assess quality of the new interpretable QA scores across the 13 considered disorder predictors, including QUARTERplus. Fig. 4 shows scatter plots of the new QA scores that quantify expected MCC values against the actual MCC values computed on the test dataset for two representative predictors: QUARTERplus and the best current method, SPOT-Disorder. We emphasize that these results do not quantify the quality of the underlying disorder predictions, which are assessed in section 3.2, but instead they focus on the quality of the QA scores that accompany these predictions. The QA scores are deemed to be useful/high-quality if they correlate with the corresponding true MCC values. It is important to note that higher correlations do not necessarily imply higher predictive performance of the corresponding disorder predictor, but rather they indicate that the QA scores are more suitable to identify accurate disorder predictions for a given predictor.

We observe that the new QA scores and the actual MCCs are correlated for both predictors in Fig. 4. The plots for the complete set of the 12 disorder predictors and QUARTERplus are in Supplementary Fig. S3 and they show similarly strong relations, except only for ESpritz‐DisProt that shows more modest correlation. The latter is due to the lower quality of the raw QA scores produced by QUARTER for ESpritz-DisProt, which we show in the corresponding panel in Supplementary Fig. S1. We assess the quality of the new QA scores (i.e., expected MCCs) by quantifying their Person correlation coefficients (PCCs) with the actual MCC scores as well as the mean absolute error (MAE) and mean squared error (MSE) between the two scores. We show these values for all 13 disorder predictors, including QUARTERplus, in Supplementary Table S2. The median (across predictors) PCC = 0.78, median MAE = 0.080 and median MSE = 0.001. This suggests that the new QA scores are highly correlated with the actual predictive performance and that the median absolute difference between the two is relatively small at 0.08, given that MCC ranges between −1 and +1.

**Fig. 5:**
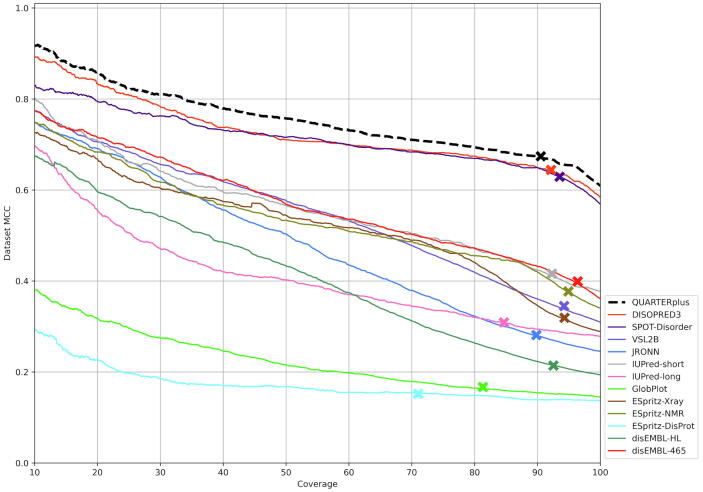
Relation between the actual MCC scores that quantify predictive performance on the independent test dataset and the new QA scores for the 13 disorder predictors, including QUARTERplus. Each line denotes the relation between actual MCC values (y-axis) for a subset of residues in the test dataset with the highest QA scores (x-axis); we call the latter the coverage by the highest QA scores and we measure it with 0.01% step size. The ‘x’ markers denote the coverage where the predictions from a given method are better by at least 10% than the results on the complete dataset and where this difference is statistically significant (*p*-value < 0.001). The statistical significance assesses whether these differences are robust to different datasets. More specifically, we sample at random 100 times 50% of residues from the complete dataset and from a given subset of the dataset (coverage value). Next, we evaluate whether these measurements are normal with the Anderson-Darling test at 0.05 significance, and we assess significance with the *t*-test for normal measurements, and with Wilcoxon rank-sum test otherwise.

Next, we apply the new interpretable QA scores in a practical context to select a subset of residue-level predictions that presumably, according to the values of the QA scores, have higher predictive quality. More specifically, we sort the predictions on the test dataset by the new QA scores and evaluate the actual MCC of the progressively smaller subsets of these predictions that have higher QA scores (Fig. 5). The 13 lines in Fig. 5 quantify the corresponding predictive performance on the test dataset for the 13 considered predictors. The lines at the top identify the most accurate disorder predictors, which include QUARTERplus, DISOPRED3 and SPOT-Disorder. We provide more detailed assessment of the disorder predictions in Section 3.2. We emphasize that Fig. 5 evaluates whether the QA scores facilitate selection of higher quality predictions for a given disorder predictor, which correspond to the predictions with higher measured MCC scores. The 13 lines in Fig. 5 are virtually monotonic, which means that the selection of residues with higher QA scores results in identifying more accurate predictions. In most cases, the predictions on the complete test dataset have much lower quality than for the subsets of amino acids selected based on QA scores. For instance, QUARTERplus secures MCC = 0.61 on the complete test dataset while its performance improves to MCC = 0.76 for the top 50% residues selected with the new QA scores. Similarly, the current predictor with the best MCC = 0.58 on the test dataset (red line in Fig. 5), DISOPRED3, obtains MCC = 0.71 for the top half of residues with highest QA scores. At the 50% coverage, we find the maximal improvement of 124% for disEMBL-HL and minimal improvement of 22% for ESpritz-DisProt when compared to their corresponding dataset-level MCC values. We also measure the coverage of the test dataset (% of residues with the highest QA scores) where the improvement in the measured MCC scores is at least 10% and statistically significant (*p*-value < 0.001). This level of coverage is indicated with the “x” marker on the plots in Fig. 5; details of the statistical tests are explained in the figure caption. The exact coverage values, the MCC improvements and the corresponding *p*-values are listed in Supplementary Table S3. Briefly, we find that median coverage of the test dataset (across the 13 predictors) with such significant improvements in MCC is 92.3%. In other words, we can use the new QA scores to select 92.3% of residues where the predictive quality is significantly higher (over 10% improvement with *p*-value < 0.001).

We analyze relation between the QA scores produced by QUARTERplus and key characteristics of disorder including size of the IDRs and proximity to IDRs. More specifically, we compare the QA values for short (<10 consecutive residues), medium (10 to 30 consecutive residues) and long (>30 consecutive residues) IDRs and compare them against the native ordered residues (Supplementary Fig. S4). This analysis reveals that the QA scores for longer IDRs are lower compared to the shorter IDRs for both versions of disEMBL and IUPred while the inverse is true for ESpritz-DisProt, VSL2B, SPOT-Disorder, DISOPRED2 and QUARTERplus. Moreover, the QA scores for the ordered residues are higher than for the disordered residues for majority of the predictors including the three versions of ESpritz, both versions of IUPred, SPOT-Disorder, DISOPRED3 and QUARTERplus. These relations suggest that the predictive quality of disorder predictors depends on the presence and size of IDRs. This observation agrees with several recent studies that similarly suggest that predictive performance is dependent on the size of IDRs and the amount of disorder in a given protein sequence [13], [31], [34], [70]. Interestingly, the lack of consistency in these relations across the predictors reveals that the QA scores are optimized to specific predictors. We also investigate whether the QA scores are sensitive to the location of the termini of IDRs. Supplementary Fig. S5 reveals a sharp change in the QA scores at these locations (i.e., for the small value of distance from the terminus) for majority of the disorder predictors. A similar observation was made in the context of the secondary structure prediction [71]. We speculate that this strong trend could be explained by possibly lower quality of the disorder annotations at these positions since that this is where we somehow arbitrarily decide which residues are flexible enough to be categorized as disordered.

### 3.2 Assessment of disorder predictions

We comparatively assess quality of the disorder predictions generated by the QUARTERplus meta-predictor against the 12 representative methods on the test dataset. The results of the top current tools reported in Table 1, SPOT-Disorder and DISOPRED3, are in line with the previous reports which used different test datasets. SPOT-Disorder was shown to secure ROC-AUC = 0.905 [58], 0.904 [34], and 0.918 [33] vs. 0.918 in our test set. Similarly, DISOPRED3 obtained ROC-AUC = 0.899 [34] and 0.897 in the CASP10 assessment [29] vs. 0.915 on our test dataset.

**Table 1:** Predictive performance of QUARTERplus and the 12 representative disorder predictors on the independent test dataset. Asterisks in the ROC-AUC, PR-AUC and MCC columns denote the fact that QUARTERplus performance is significantly higher than a given other disorder predictor (*p*-value < 0.001). The statistical significance assesses whether these differences are robust to different datasets. More specifically, we measure predictive quality for 100 repetitions of tests on 50% of randomly selected test proteins. Next, we evaluate whether these measurements are normal with the Anderson-Darling test at 0.05 significance, and we assess significance with the *t*-test for normal measurements, and with Wilcoxon rank-sum test otherwise. The predictors are sorted by their ROC-AUC values. Best results are denoted by bold font.

Predictor	ROC-AUC	PR-AUC	MCC
QUARTERplus	**0.929**	**0.647**	**0.611**
SPOT‐Disorder	0.918*	0.582*	0.569*
DISOPRED3	0.915*	0.619*	0.584*
VSL2B	0.839*	0.389*	0.309*
ESpritz-Xray	0.812*	0.355*	0.289*
IUPred‐short	0.810*	0.382*	0.377*
ESpritz-NMR	0.807*	0.364*	0.340*
disEMBL-465	0.804*	0.357*	0.361*
ESpritz-DisProt	0.782*	0.167*	0.137*
JRONN	0.766*	0.306*	0.246*
disEMBL-HL	0.760*	0.255*	0.194*
IUPred‐long	0.732*	0.265*	0.278*
GlobPlot	0.630*	0.139*	0.145*

Table 1 reveals that the predictive performance of QUARTERplus measured with ROC-AUC, PR-AUC and MCC is significantly higher than the performance of the other 12 predictors (*p*-value < 0.001). The predictions produced by our novel meta architecture secure very high ROC-AUC = 0.929 and are highly correlated with the native annotations of disorder, with MCC = 0.611. The relative reduction of error reported with AUC-ROC between QUARTERplus and the second-best SPOT-Disorder is (0.929–0.918)/(1–0.918) = 13%. The median increase in AUC-ROC when comparing QUARTERplus with the other predictors is 0.123. We secure similarly large improvements when using MCC to assess binary predictions. For instance, the median increase in MCC when comparing with the 12 other predictors is 0.312, with the 0.03 improvement over the second best MCC secured by DISOPRED3. Overall, we conclude that QUARTERplus generates very accurate disorder predictions that statistically outperform the current predictors, including the three tools that are used as inputs to our meta-method. The rationale behind these improvements is the ability of the deep recurrent network to use the QA scores to guide the consensus prediction. In other words, the QA scores associated with the three input disorder predictions are used to identify problematic input predictions to better inform computation of the output meta-prediction.

### 3.3 Case study

**Fig. 6:**
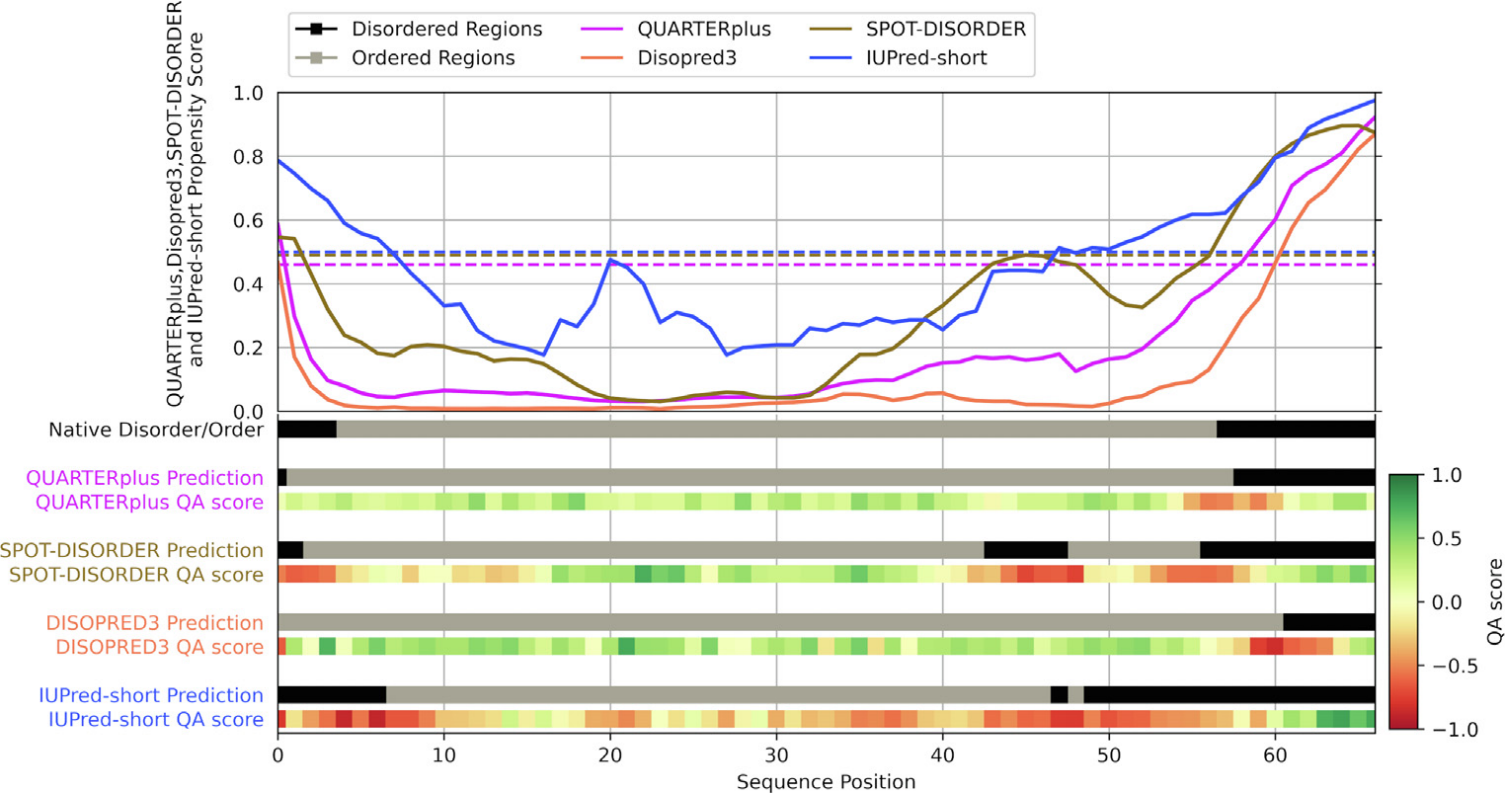
Predictions from QUARTERplus and its input/base predictors (SPOT-Disorder, DISOPRED3 and IUPred-short) for a test protein, 50S ribosomal protein L24e. Plots at the top represent disorder propensity scores that are color-coded by predictor. The horizontal lines at the bottom represent native disorder annotations and the corresponding putative binary predictions, with black for disorder and gray for order. They are accompanied by the color-coded interpretable QA scores. The color map for the QA scores ranges from dark red (strongly negative QA scores) to dark green (strongly positive QA scores). (For interpretation of the references to color in this figure legend, the reader is referred to the web version of this article.)

We discuss and explain the QUARTERplus outputs for one of test proteins, the 50S ribosomal protein L24e (Uniprot id: P14116) from *Haloarcula marismortui*, a halophilic Archaeon. This case study is not intended to quantify expected predictive quality but rather to explain the QUARTERplus outputs and relate them to the input predictions from DISOPRED3, SPOT-Disorder and IUPred-short. We secure the native disorder annotations from the crystal structure (PDB ID: 3CC2) which includes disordered regions at both termini (positions 1–4 and positions 57–66). The top of Fig. 6 illustrates the disorder propensity scores produced by QUARTERplus (in purple), SPOT‐Disorder (brown), DISOPRED3 (orange) and IUPred‐short (blue). The bottom of Fig. 6 shows native disorder annotations and the corresponding putative binary predictions (black for disorder and gray for order) accompanied by the color-coded interpretable QA scores. The color map for the QA scores ranges from dark red (strongly negative QA scores) to dark green (strongly positive QA scores).

The input disorder predictors generate relatively accurate results with MCC = 0.61 (SPOT‐Disorder), 0.61 (DISOPRED3), and 0.65 (IUPred‐short). SPOT‐Disorder predicts disorder at both termini but also includes a false positive region between positions 44 and 48. DISOPRED3 predicts only one disordered region at the C terminus with a few false negatives (underpredicted disorder). IUPred‐short over-predicts disorder at both termini. Importantly, the incorrect predictions generated by these tools are flagged with red QA scores that suggest high likelihood of error. For instance, SPOT-Disorder prediction at the N-terminus is flagged red since it underpredicts disorder there, while the over-predicted disordered region in the middle of the sequence is also marked red. The QUARTERplus meta-predictor improves over the input predictions, which is consistent with the overall test results, and secures MCC = 0.81. Its QA scores are mostly green correctly suggesting that these predictions are likely accurate. The only exception is the orange-colored edge of the predicted disordered region at the C-terminus where QUARTERplus indeed under-predicts disorder. The meta-predictor succeeds in correctly adjusting results produced by its three input predictions since it has access to the corresponding three sets of QA scores that point to regions where these inputs are possibly incorrect. This demonstrates the underlying value of the new QA scores that help to build a better meta model while also allowing the users to evaluate its outputs.

### 3.4 QUARTERplus webserver

We provide convenient access the QUARTERplus method, including the meta-predictor of disorder and interpretable QA scores, at http://biomine.cs.vcu.edu/servers/QUARTERplus/. This webserver also produces the disorder predictions and the associated QA scores for several other popular methods: SPOT-Disorder, DISOPRED3, IUPred-short, IUPred-long, VSL2B, disEMBL-HL, disEMBL-465 and GlobPlot. We exclude ESpritz since its authors disallow inclusion of this software into derived predictive platforms.

QUARTERplus webserver takes the FASTA-formatted amino acid sequence(s) of the input protein(s) as the only input. We service batch predictions of up to 50 sequences per request for the faster predictors (IUPred-short, IUPred-long, VSL2B, disEMBL-HL, disEMBL-465 and GlobPlot) and single-sequence requests for the slower tools (QUARTERplus, SPOT-Disorder and DISOPRED3). We process the requests using a queue that serves multiple webservers from our lab and which ensures load balancing between users. The entire process, including prediction of disorder and production of the QA scores, is automated and performed on the server side. This frees the user from using their own hardware. The end results include the disorder predictions including binary and propensity scores that are accompanied by the QA scores. We deliver the results in the browser window and inform the users via email (if email address was provided) when the prediction completes. We provide the results for the fast predictors in parsable csv-formatted file. We generate the results for the slower and more accurate methods, including QUARTERplus, in two ways: as the parsable csv file and as high-quality graphic (example shown in Fig. 6) directly in the web browser window.

## 4 Summary

Prediction of intrinsic disorder from protein sequences is a long standing and well-researched topic [11], [28], [29], [30], [31], [32], [10], [33], [34], [72]. A new aspect that recently gained attention is the development of QA tools that provide useful, residue-level clues concerning quality of the disorder predictions [35], [36]. However, the only current QA tool, QUARTER, suffers several substantial drawbacks. We address these issues with the new QUARTERplus method.

QUARTERplus is an innovative deep learning meta-model that delivers highly accurate disorder predictions by combining QA scores and the associated disorder predictions from three modern input predictors. We empirically demonstrate that QUARTERplus’s predictions are statistically more accurate than the results generated by a representative set of twelve modern disorder predictors, including highly-ranked methods from the CASP10 and CAID community assessments [29], [33], [73]. More importantly, these accurate predictions are accompanied by easy to interpret residue-level QA scores that reliably quantify their predictive quality. We provide this feature for QUARTERplus and several other state-of-the-art disorder predictors. These new QA scores are highly correlated with the actual/measured predictive performance quantified with MCC, which means that they effectively identify regions of correct vs. incorrect disorder predictions. For instance, while QUARTERplus produces disorder predictions with the overall MCC of 0.61, using the QA scores we are able to identify the better half of these predictions where MCC is 0.76. We also provide a convenient webserver for QUARTERplus at http://biomine.cs.vcu.edu/servers/QUARTERplus/. To sum up, QUARTERplus is a convenient tool that provides highly accurate disorder predictions, empowers the users to easily pinpoint which predictions are more trustworthy with the help of the easy to interpret QA scores, and generates these scores for several popular disorder predictors.

## Funding

This work was supported in part by the National Science Foundation (grant 1617369) and the Robert J. Mattauch Endowment funds to L.K.

## CRediT authorship contribution statement

**Akila Katuwawala:** Conceptualization, Data curation, Methodology, Validation, Software, Writing - original draft, Writing - review & editing. **Sina Ghadermarzi:** Validation, Software, Writing - review & editing. **Gang Hu:** Conceptualization, Writing - review & editing. **Zhonghua Wu:** Conceptualization, Writing - review & editing. **Lukasz Kurgan:** Conceptualization, Validation, Writing - original draft, Writing - review & editing, Project administration, Supervision, Funding acquisition.

## Declaration of Competing Interest

The authors declare that they have no known competing financial interests or personal relationships that could have appeared to influence the work reported in this paper.
