# Supplementary material for: QUARTERplus: Accurate disorder predictions integrated with interpretable residue-level quality assessment scores
Source: Comput Struct Biotechnol J. 2021 Apr 27;19:2597–606. doi: 10.1016/j.csbj.2021.04.066 (PMC8122155; doi:10.1016/j.csbj.2021.04.066)
Supplement: Supplementary data 1 [file mmc1.pdf]

SUPPLEMENT

Supplementary Tables

Supplementary Table S1. Deep network architectures considered during the optimization of the hyper-parameters. For each of the 21 considered topologies we specify the type of layers and the number of neurons per layer. Abbreviations: FFN (feed forward network), LSTM (long short-term memory network) and BID (bidirectional long short-term memory network).

FFN 1		FFN 2		FFN 3		FFN 4		FFN 5		FFN 6		FFN 7	
Layer	Neurons	Layer	Neurons	Layer	Neurons	Layer	Neurons	Layer	Neurons	Layer	Neurons	Layer	Neurons
Dense	200	Dense	100	Dense	50	Dense	25	Dense	12	Dense	6	Dense	3
Dropout	0.5	Dropout	0.5	Dropout	0.5	Dropout	0.5	Dropout	0.5	Dropout	0.5	Dropout	0.5
Dense	200	Dense	100	Dense	50	Dense	25	Dense	12	Dense	6	Dense	3
Dropout	0.5	Dropout	0.5	Dropout	0.5	Dropout	0.5	Dropout	0.5	Dropout	0.5	Dropout	0.5
Dense	64	Dense	100	Dense	50	Dense	25	Dense	12	Dense	6	Dense	3
Dropout	200	Dropout	0.5	Dropout	0.5	Dropout	0.5	Dropout	0.5	Dropout	0.5	Dropout	0.5
Dense	64	Dense	100	Dense	50	Dense	25	Dense	12	Dense	6	Dense	3
Dropout	200	Dropout	0.5	Dropout	0.5	Dropout	0.5	Dropout	0.5	Dropout	0.5	Dropout	0.5
Dense	1	Dense	1	Dense	1	Dense	1	Dense	1	Dense	1	Dense	1

LSTM 1		LSTM 2		LSTM 3		LSTM 4		LSTM 5		LSTM 6		LSTM 7	
Layer	Neurons	Layer	Neurons	Layer	Neurons	Layer	Neurons	Layer	Neurons	Layer	Neurons	Layer	Neurons
Dense	10	Dense	10	Dense	10	Dense	10	Dense	10	Dense	10	Dense	5
LSTM	200	LSTM	100	LSTM	50	LSTM	25	LSTM	12	LSTM	6	LSTM	3
LSTM	200	LSTM	100	LSTM	50	LSTM	25	LSTM	12	LSTM	6	LSTM	3
Dense	1	Dense	1	Dense	1	Dense	1	Dense	1	Dense	1	Dense	1

BID 1		BID 2		BID 3		BID 4		BID 5		BID 6		BID 7	
Layer	Neurons	Layer	Neurons	Layer	Neurons	Layer	Neurons	Layer	Neurons	Layer	Neurons	Layer	Neurons
3d_Dense	10	3d_Dense	10	3d_Dense	10	3d_Dense	10	3d_Dense	10	3d_Dense	10	3d_Dense	5
Bi_LSTM	200	Bi_LSTM	100	Bi_LSTM	50	Bi_LSTM	25	Bi_LSTM	12	Bi_LSTM	6	Bi_LSTM	3
Dropout	0.5	Dropout	0.5	Dropout	0.5	Dropout	0.5	Dropout	0.5	Dropout	0.5	Dropout	0.5
3d_Dense	10	3d_Dense	10	3d_Dense	10	3d_Dense	10	3d_Dense	10	3d_Dense	10	3d_Dense	5
Bi_LSTM	200	Bi_LSTM	100	Bi_LSTM	50	Bi_LSTM	25	Bi_LSTM	12	Bi_LSTM	6	Bi_LSTM	3
Dropout	0.5	Dropout	0.5	Dropout	0.5	Dropout	0.5	Dropout	0.5	Dropout	0.5	Dropout	0.5
3d_Dense	10	3d_Dense	10	3d_Dense	10	3d_Dense	10	3d_Dense	10	3d_Dense	10	3d_Dense	5
Bi_LSTM	200	Bi_LSTM	100	Bi_LSTM	50	Bi_LSTM	25	Bi_LSTM	12	Bi_LSTM	6	Bi_LSTM	3
Dropout	0.5	Dropout	0.5	Dropout	0.5	Dropout	0.5	Dropout	0.5	Dropout	0.5	Dropout	0.5
Dense	1	Dense	1	Dense	1	Dense	1	Dense	1	Dense	1	Dense	1

Supplementary Table S2. Pearson Correlation coefficients (PCC), Mean Squared Error (MSE) and Mean Absolute Error (MAE) between expected the QA scores produced by QUARTERplus that quantify expected MCCs and the actual MCCs measured on the independent test dataset. We show results for 12 representative disorder predictors and the QUARTERplus methods. The predictors are sorted by the PCC of their new QA scores.

Predictor	PCC	MSE	MAE
VSL2B	0.95	0.001	0.055
JRONN	0.95	0.001	0.053
disEMBL-HL	0.94	0.001	0.051
disEMBL-465	0.89	0.001	0.069
ESpritz-Xray	0.85	0.001	0.085
IUPred-short	0.80	0.001	0.080
SPOT-Disorder	0.78	0.001	0.073
ESpritz-NMR	0.78	0.001	0.087
DISOPRED3	0.77	0.001	0.092
GlobPlot	0.74	0.001	0.061
IUPred-long	0.68	0.001	0.089
QUARTERplus	0.64	0.001	0.101
ESpritz-DisProt	0.32	0.002	0.097

Supplementary Table S3. The coverage of the independent test dataset that gives at least 10% improvement in measured MCC values where this difference is statistically significant (p-value < 0.001). The statistical significance assesses whether these differences are robust to different datasets. More specifically, we sample at random 100 times 50% of residues from the complete dataset and from a given subset of the dataset (coverage value). Next, we evaluate whether these measurements are normal with the Anderson-Darling test at 0.05 significance, and we assess significance with the t-test for normal measurements, and with Wilcoxon rank-sum test otherwise.

Predictor	Amount of improvement in MCC for the selected predictions [%]	Coverage by the selected predictions [%]	*p*-value when comparing selected predictions against the complete test dataset
disEMBL-465	11.6	96.4	<0.001
ESpritz-NMR	11.0	94.9	<0.001
ESpritz-Xray	10.4	94.3	<0.001
VSL2B	11.6	94.2	<0.001
SPOT-Disorder	10.6	93.5	<0.001
disEMBL-HL	10.6	92.5	<0.001
IUPred-short	10.3	92.3	<0.001
DISOPRED3	10.4	92.2	<0.001
QUARTERplus	10.3	90.5	<0.001
JRONN	14.2	89.8	<0.001
IUPred-long	10.9	84.7	<0.001
GlobPlot	11.9	81.3	<0.001
ESpritz-DisProt	18.4	71.0	<0.001

Supplementary Figures

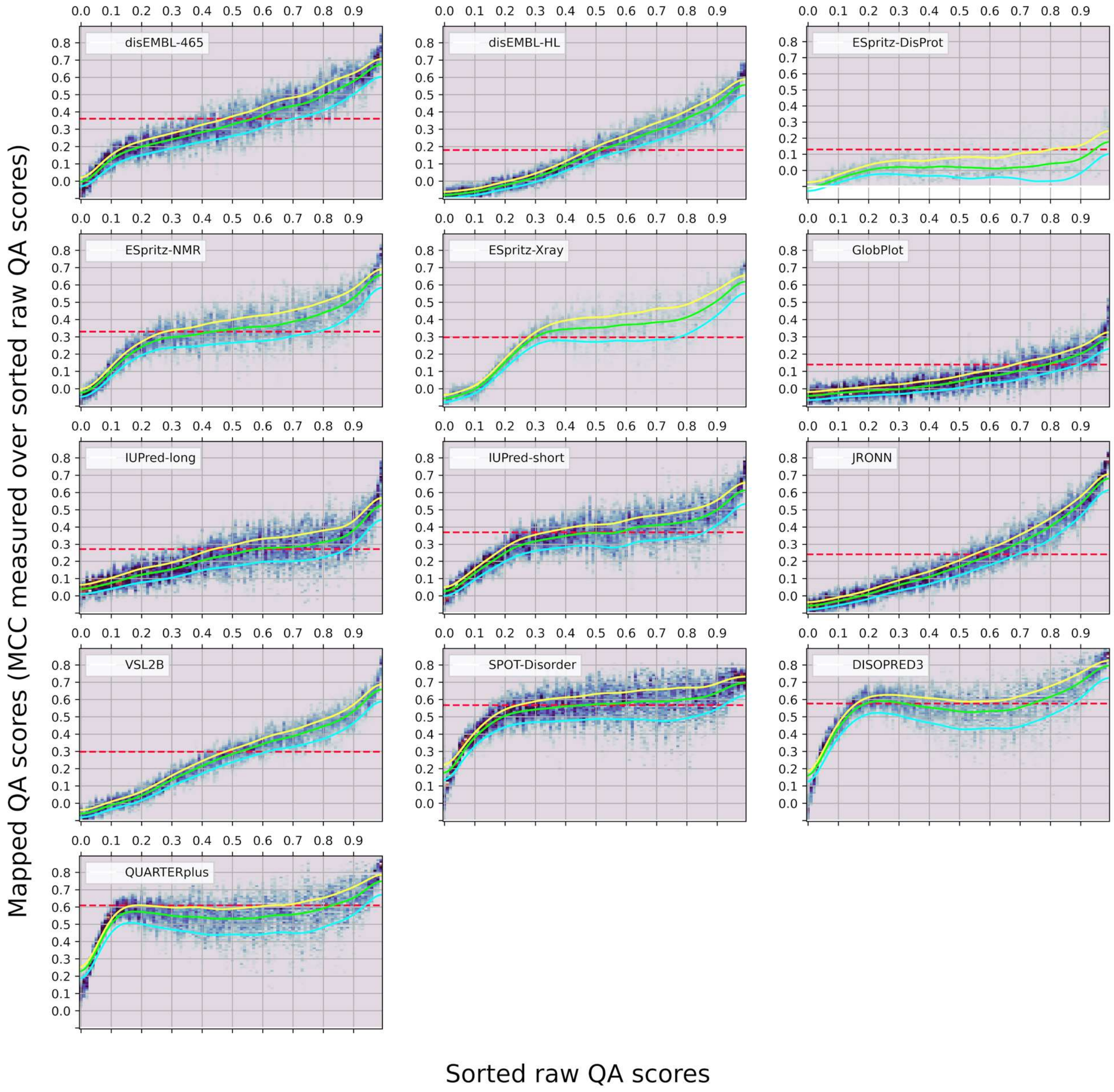

Supplementary Figure S1. MCC distributions that we use to design the MCC mapping functions that transform the raw QUARTER-produced QA scores into the interpretable QA scores. The yellow, green and blue lines correspond to 75th, 50th and 25th percentile of the MCC scores computed using the sliding window over the sorted raw QA scores in the training dataset. The red horizontal dashed line is the MCC obtained on the training dataset.

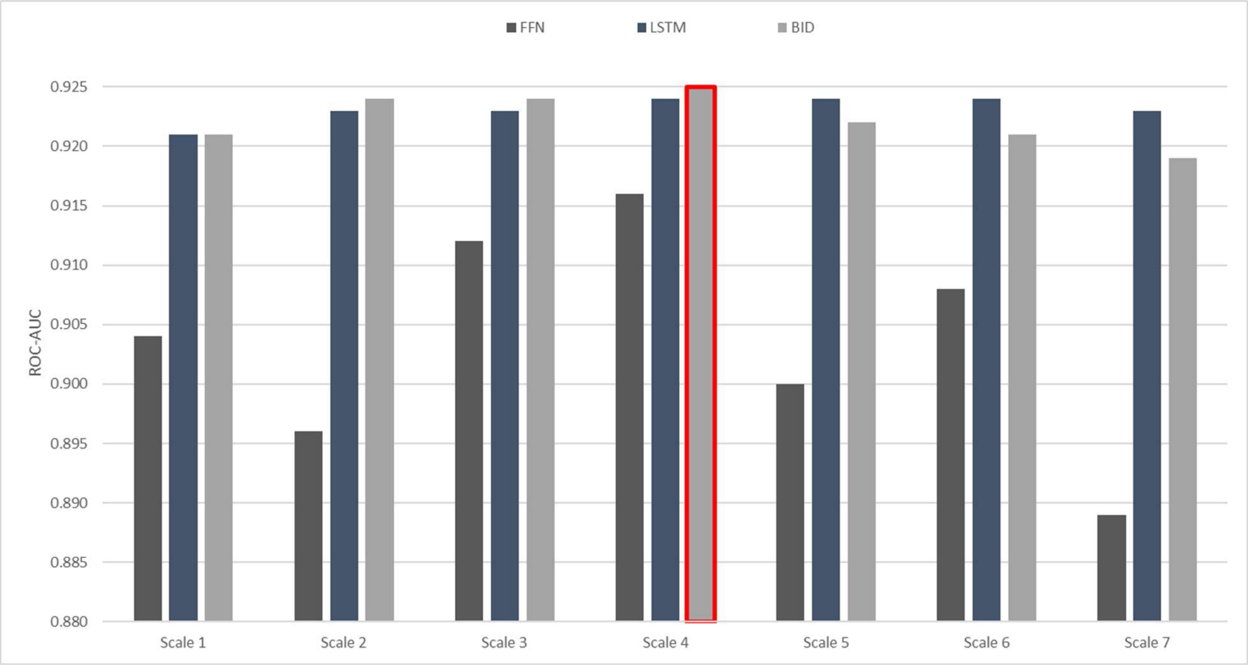

Supplementary Figure S2. Predictive performance quantified with ROC-AUC measured on the validation set for the 21 considered designs of the deep networks detailed in Supplementary Table S1. The red edge identifies the selected best design. Abbreviations: FFN (feed forward network), LSTM (long short-term memory network) and BID (bidirectional long short-term memory network). Scale value identifies the network sizes defined in Supplementary Table S1.

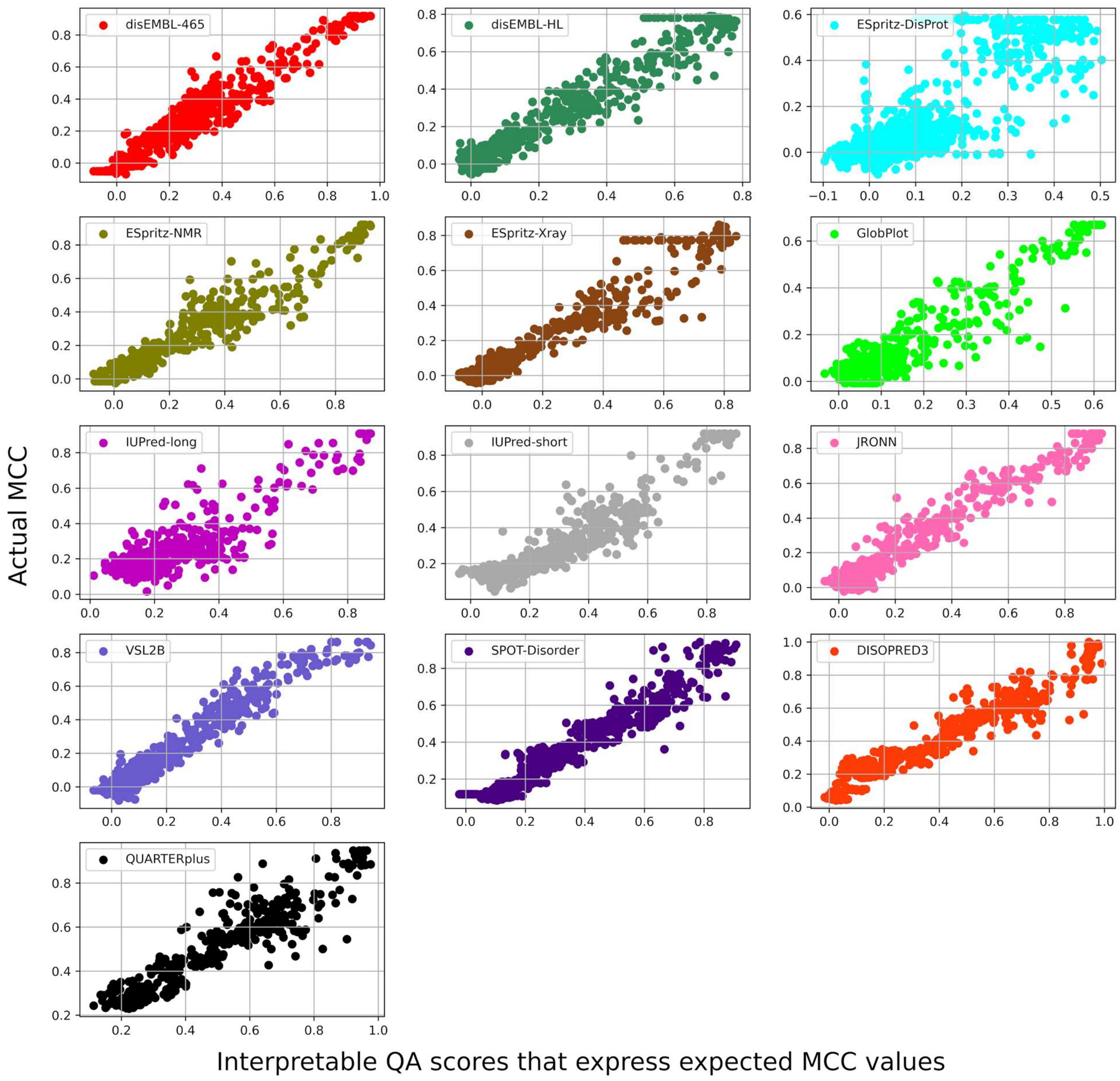

Supplementary Figure S3. Scatter plots of the actual MCC values (*y*-axis) against the new QA scores that express expected MCC values (*x*-axis) computed on the independent test dataset for the 12 disorder predictors and QUARTERplus. We use the sliding windows of the residues sorted by the QUARTER scores to produce the actual MCC scores (details in Section 2.5).

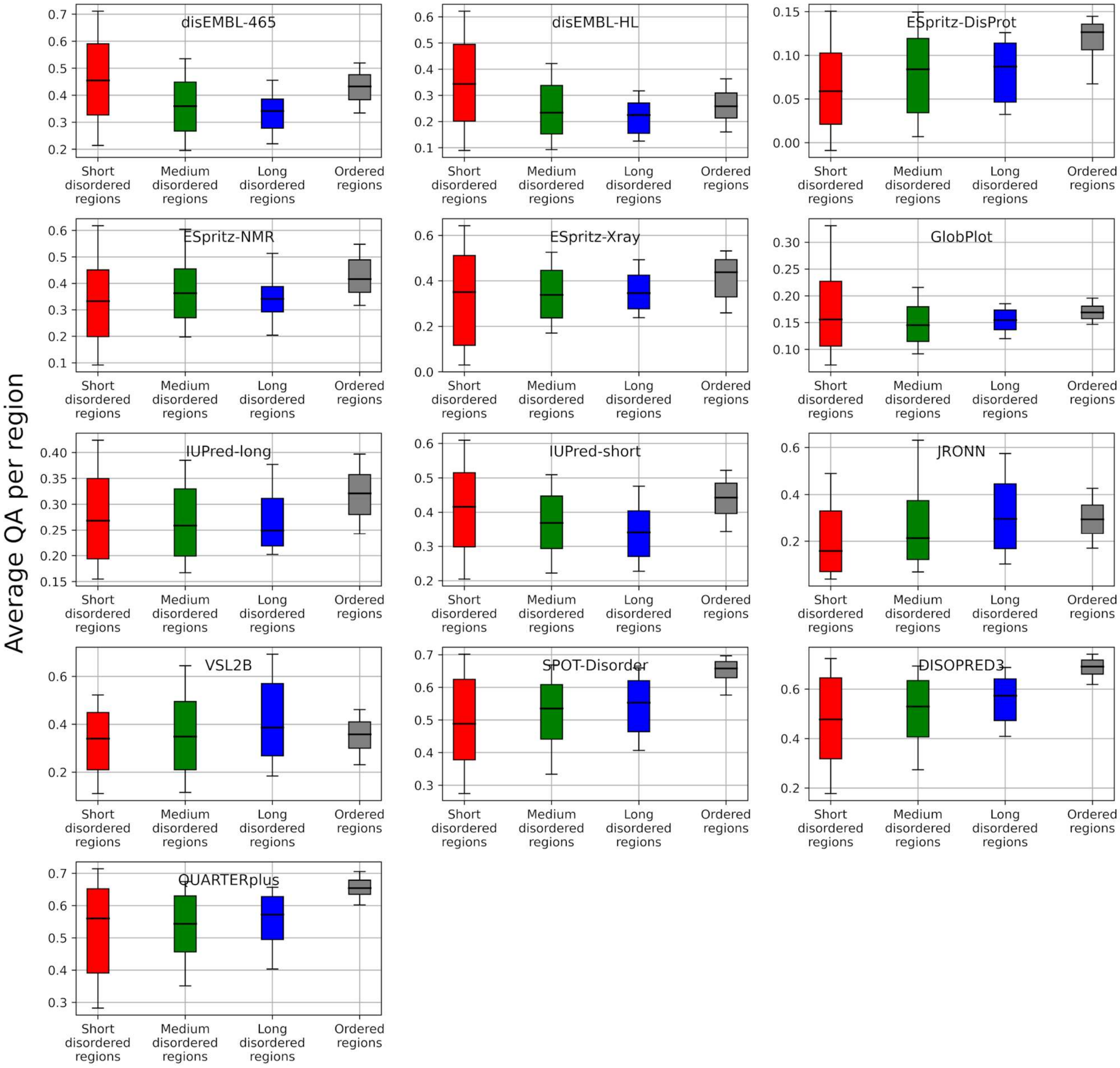

Supplementary Figure S4. Distributions of the QA scores produced by QUARTERplus for the native ordered residues and short (below 10 consecutive residues), medium (10 to 30 consecutive residues) and long (over 30 consecutive residues) IDRs in the independent test dataset. Box plots show the 25th, 50th (median) and 25th percentile of the MCC scores while the error bars identify the minimal and maximal MCC values. Individual panels present this analysis for each of the 12 disorder predictors and QUARTERplus.

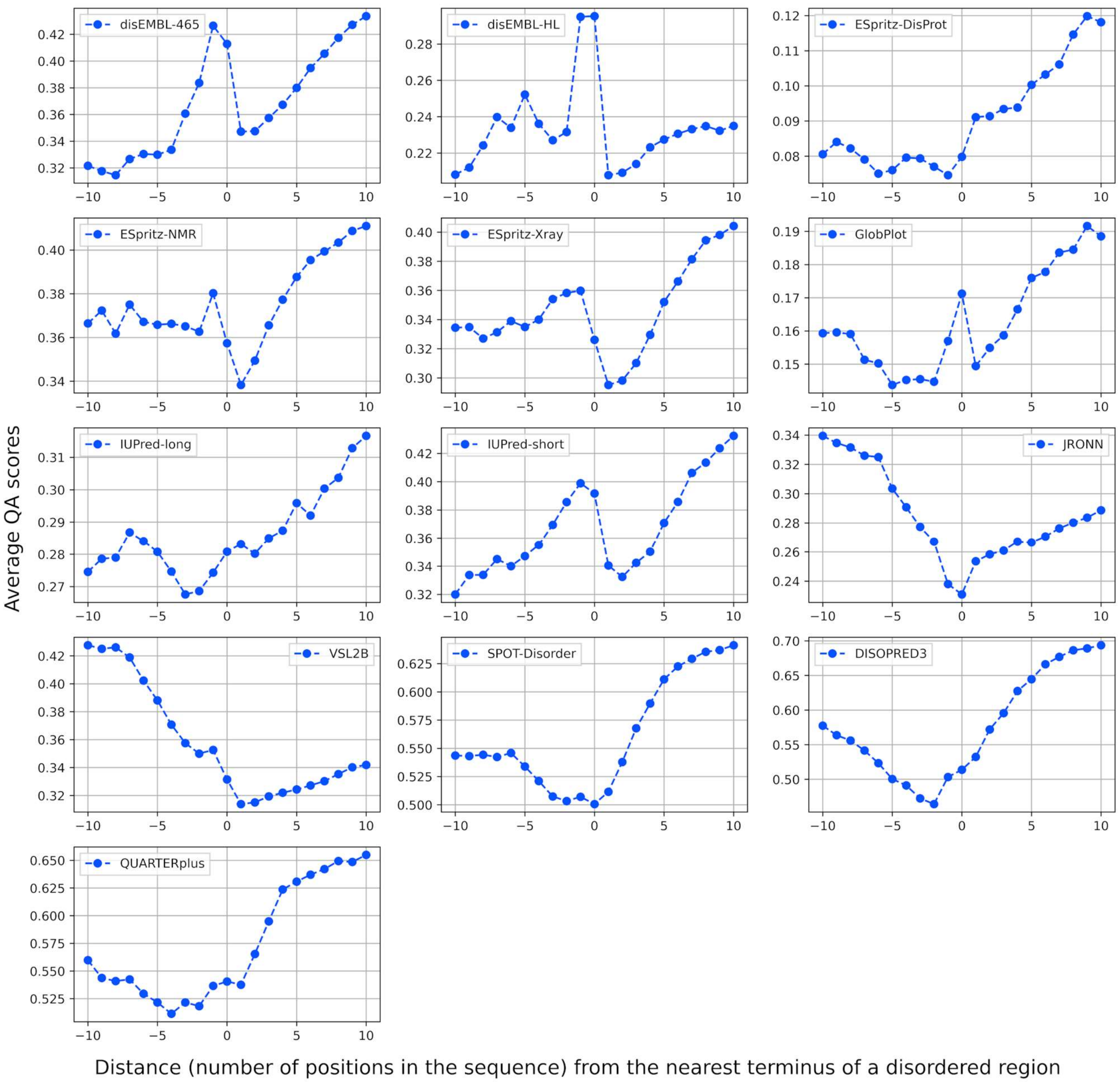

Supplementary Figure S5. Average QA scores produced by QUARTERplus for residues in the vicinity of the terminus of an IDR. The *x*-axis shows the distance measured as the number of positions in the sequences to the nearest terminus of an IDR. Positive values of *x* denote residues in the disordered regions while negative values of *x* denote residues in disordered regions. Value of 0 denotes residues that are adjacent to the terminus of an IDR. Individual panels present this analysis for each of the 12 disorder predictors and QUARTERplus.
